# Clinical and genetic analysis of *ATP13A2* in hereditary spastic paraplegia expands the phenotype

**DOI:** 10.1002/mgg3.1052

**Published:** 2020-01-15

**Authors:** Mehrdad A. Estiar, Etienne Leveille, Dan Spiegelman, Nicolas Dupre, Jean-François Trempe, Guy A. Rouleau, Ziv Gan‐Or

**Affiliations:** ^1^ Department of Human Genetics McGill University Montréal QC Canada; ^2^ Montreal Neurological Institute and Hospital McGill University Montréal QC Canada; ^3^ Faculty of Medicine McGill University Montréal QC Canada; ^4^ Department of Neurology and Neurosurgery McGill University Montréal QC Canada; ^5^ Department of Medicine Faculty of Medicine Université Laval Quebec City QC Canada; ^6^ Department of Pharmacology & Therapeutics McGill University Montréal QC Canada; ^7^ Centre for Structural Biology McGill University Montréal QC Canada

**Keywords:** *ATP13A2*, HSP, Neurodegeneration, Parkinsonism

## Abstract

**Background:**

Hereditary spastic paraplegias (HSP) are neurodegenerative disorders characterized by lower limb spasticity and weakness, with or without additional symptoms. Mutations in *ATP13A2,* known to cause Kufor–Rakeb syndrome (KRS), have been recently implicated in HSP.

**Methods:**

Whole‐exome sequencing was done in a Canada‐wide HSP cohort.

**Results:**

Three additional patients with homozygous *ATP13A2* mutations were identified, representing 0.7% of all HSP families. Spastic paraplegia was the predominant feature, all patients suffered from psychiatric symptoms, and one patient had developed seizures. Of the identified mutations, c.2126G>C;(p.[Arg709Thr]) is novel, c.2158G>T;(p.[Gly720Trp]) has not been reported in ATP13A2‐related diseases, and c.2473_2474insAAdelC;p.[Leu825Asnfs*32]) has been previously reported in KRS but not in HSP. Structural analysis of the mutations suggested a disruptive effect, and enrichment analysis suggested the potential involvement of specific pathways.

**Conclusion:**

Our study suggests that in HSP patients with psychiatric symptoms, *ATP13A2* mutations should be suspected, especially if they also have extrapyramidal symptoms.

## Graphical abstract

In this study, WES in a large Canadian cohort of HSP patients identified three patients with biallelic mutations in ATP13A2. The mutations, their potential effects on the protein structure and the clinical presentation of the patients are being discussed.

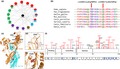

## 1 INTRODUCTION

Hereditary spastic paraplegia (HSP) is a group of neurodegenerative disorders characterized by lower limb spasticity and weakness, with or without additional symptoms (Faber, Pereira, Martinez, França Jr, & Teive, 2017). Some HSP‐related genes may be involved in other disorders in which spasticity is not among the main features, for example, *FA2H* (OMIM 611026) and *KIAA1840* (OMIM 610844) mutations may cause neurodegeneration with brain‐iron accumulation and Charcot–Marie–Tooth (CMT) (Kruer et al., 2010; Montecchiani et al., 2015). Similarly, genes that are involved in other neurological disorders, such as *ALS2* (OMIM 205100) and *POLR3A* (OMIM 614258), were also implicated in HSP (Eymard‐Pierre et al., 2002; Rydning et al., 2019).

One of the most interesting genes in the latter category is *ATP13A2* (OMIM 610513), which was initially implicated in Kufor–Rakeb syndrome (KRS, OMIM 606693), characterized by early onset parkinsonism, pyramidal tract degeneration, dementia, and cognitive dysfunction (Ramirez et al., 2006). Subsequently, *ATP13A2* mutations were reported in Neuronal Ceroid Lipofuscinosis (NCL) and amyotrophic lateral sclerosis (ALS; Farias et al., 2011; Spataro et al., 2019). *ATP13A2* mutations in HSP (SPG78, OMIM 617225) were first described in a consanguineous Pakistani family (Kara et al., 2016), followed by three reports on five more families (Erro, Picillo, Manara, Pellecchia, & Barone, 2019; Estrada‐Cuzcano et al., 2017; van de Warrenburg et al., 2016). *ATP13A2* encodes a lysosomal enzyme which serves as an inorganic cation transporter that regulates endolysosomal cargo sorting and neuronal integrity (Demirsoy et al., 2017; Ramonet et al., 2011).

Herein, we report three additional HSP patients from three different families with homozygous *ATP13A2* mutations. Long‐term follow‐up, genetic analysis, protein structure, and network analyses were done to explore the clinical and genetic spectrum of *ATP13A2*‐related disease.

## 2 METHODS

### 2.1 Population

HSP patients (*n* = 696) from 431 families were recruited across Canada, and data on diagnosis, recruitment, and the cohort were previously published (Chrestian et al., 2017). Of those, 383 HSP genetically undiagnosed patients went through whole‐exome sequencing (WES). All participants have signed an informed consent form and the study protocol was approved by the institutional review board.

### 2.2 Genetic analysis

Whole‐exome capture, sequencing, alignment, annotation, and variant calling was performed as previously described (Chrestian et al., 2017). Nonsynonymous, frameshift, splice‐site, and stop variants with allele frequencies <0.005 in the Exome Aggregation Consortium (ExAC) database were filtered‐in, and segregation analysis was performed. The potential pathogenicity of variants was estimated based on their frequency of in gnomAD and ExAC, and by in silico tools: MutationTaster, combined annotation‐dependent depletion (CADD), genomic‐evolutionary rate profiling (GERP++), sorting intolerant from tolerant (SIFT), and PolyPhen‐2.

### 2.3 In silico analysis of ATP13A2

Genic intolerance of *ATP13A2* was assessed using the residual variation intolerance score (RVIS) tool. Pathways enrichment and interaction network were analyzed using GeneMANIA, g:Profiler, and STRING, and networks were visualized by Cytoscape. Clustal Omega program was used for protein sequence alignment of multiple species. A 3D atomic model of human ATP13A2 was built using the automated I‐TASSER server. The steric clashes induced by each mutation were evaluated using the mutagenesis toolbox in PyMol v.2.2.0.

## 3 RESULTS

### 3.1 *ATP13A2* mutations are responsible for 0.7% of families with HSP in Canada and may affect the protein structure and function

**Figure 1:**
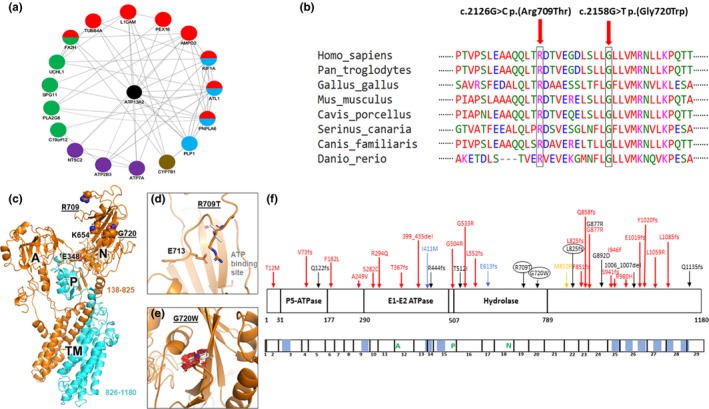
In silico analysis of ATP13A2. (a) Network analysis demonstrated that ATP13A2 is associated with other HSP‐related proteins. Green: putative homologs are comentioned or coexpressed in other species, purple: shared protein domains, brown: genetic interactions, blue: colocalization, red: coexpression. (b) Conservation of the residues harboring missense mutations in the ATP13A2 protein. (c) Cartoon representation of human ATP13A2 a.a. 138–1180. The position of the cytosolic A‐, P‐, and N‐domains, and transmembrane (TM) helices are indicated. Lys654 is an invariant lysine that interacts with the adenine ring of ATP prior to the g‐phosphate transfer. Glu348 is the catalytic glutamate in the invariant TGE motif. The HSP mutation sites p.(Arg709Thr) and p.(Gly720Trp) are underlined. The segment consisting of a.a. 826–1180 (cyan) would be deleted in the p.(Leu825Asnfs*32) mutation. (d) Arg709 is located in the N‐domain, on the opposite side of the ATP‐binding site. The mutation p.(Arg709Thr) would result in the loss of a favorable electrostatic interaction, which would destabilize the N‐domain. (e) Gly720 is located in the middle of a β‐strand in the N‐domain. The mutation p.(Gly720Trp) would create significant steric clashes (red), thus likely unfolding the N‐domain. (f) Schematic representation of the location of *ATP13A2* mutations in HSP, ALS, KRS, and NCL patients reported so far (Park et al., 2015; Spataro et al., 2019). The top schematic represents the ATP13A2 protein. Functional domains, including the P‐5 ATPase, E1‐E2 ATPase, and hydrolase domains, are indicated with vertical lines. Mutations associated with HSP are indicated in black (mutations identified in this study are circled), ALS in blue, KRS in red, and NCL in yellow. The bottom schematic represents the cDNA of *ATP13A2*. Exons are delineated with vertical line, and the location of the transmembrane domains are colored in blue

Biallelic homozygous *ATP13A2* mutations were identified in three patients (representing 0.4% of HSP patients and 0.7% of families), including c.2473_2474insAAdelC;p.(Leu825Asnfs*32), c.2126G>C;p.(Arg709Thr), and c.2158G>T;p.(Gly720Trp). The p.(Leu825Asnfs*32) and p.(Arg709Thr) variants were not reported in gnomAD (https://gnomad.broadinstitute.org), and the p.(Gly720Trp) variant has a very low allele frequency of 0.000026 in Europeans in gnomAD. *ATP13A2* is highly intolerable for functional genetic variations with an RVIS score of −1.16, putting it in the top 6.1% of intolerant human genes. Interaction network analysis (Figure 1a) demonstrated that the ATP13A2 protein closely interacts with other HSP‐related proteins. Pathway enrichment analysis of genes which are known to be involved in HSP, ALS, and Parkinsonism showed enrichment (FDR *p* < .05, Table S1) of genes involved in copper ion binding, vesicle‐mediated transport cellular response to oxidative stress, among others. Both p.(Arg709Thr) and p.(Gly720Trp) destabilize the N‐domain of ATP13A2 protein and are conserved (Figure 1b–e) and the p.(Leu825Asnfs*32) mutation deletes an entire segment at the C‐terminal of the protein (Figure 1c–e). The distribution of the current and previously reported mutations in *ATP13A2* in HSP, KRS, ALS, and NCL (Park, Blair, & Sue, 2015; Spataro et al., 2019) is depicted in Figure 1f.

### 3.2 Clinical characteristics of hsp patients with *ATP13A2* mutations

Table 1 details the clinical characteristics of previously published *ATP13A2*‐related HSP patients and the three patients identified in this study. The description of the patients below will detail only the main characteristics.

**Table 1:** Characteristics of HSP patients harboring *ATP13A2* mutations

Nucleotide change	AA change	Inheritance	Clinical Signs	MRI	Age at Onset/Sex	Origin	Reference
c.3017_3019del	p.Leu1006_Leu1007del	H	Spastic quadriplegia, falls, cognitive decline, pes cavus, ataxia, bilateral divergent squints, nystagmus on lateral gaze, reduced upgaze, No parkinsonian features	Cerebral atrophy and subtle abnormalities of the basal ganglia	18/M	Pakistani	Kara et al. (2016)
c.2675G>A	p.Gly892Asp	H	Spastic tetraplegia, cognitive decline, upgaze limitation, slow vertical saccades, mild Parkinsonism, gait abnormality, speech and swallowing difficulties, dysarthria, jerky eye movements, weakness and atrophy of the tongue, thoracic scoliosis, upper limb rigidity, bradykinesia on finger‐tapping	Cerebral and cerebellar atrophy	11/M	Dutch	van de Warrenburg et al. (2016)
c.1535C>T	p.Thr512Ile	H	Lower limb spasticity, lower limb weakness, upper and lower limb hyperreflexia, Babinski sign, Oculomotor disturbance, dysarthria, limb ataxia, slight verbal memory deficit, surface sensation deficit, vibration deficit, mild cognitive impairment, cerebellar ataxia, and axonal motor and sensory polyneuropathy	Cerebellar > cortical atrophy, Periventricular white matter changes, ear of the lynx sign	30/M, 33/M, 30/M	Bulgarian	Estrada‐Cuzcano et al. (2017)
c.364C>T	p.Gln122Ter	H	Spastic paraplegia, Lower limb spasticity, lower limb weakness, upper and lower limb hyperreflexia, neurogenic bladder dysfunction, mild dysarthria, severe dementia, labile motivation, Oculomotor disturbance, limb ataxia, vertical supranuclear gaze palsy, urge incontinence, mixed axonal‐demyelinating motor polyneuropathy	Cerebellar > cortical/mesencephalic atrophy, thin corpus callosum, hydrocephalus, periventricular white matter changes	36/F	Serbian	Estrada‐Cuzcano et al. (2017)
c.1330C>T/3403C>T	p.Arg444Ter/Gln1135Ter	C	Lower limb spasticity, upper and lower limb weakness, upper and lower limb hyperreflexia, Babinski signs, severe fronto‐temporal dementia, aggression, acoustic hallucinations, Bradykinesia, resting tremor, oculomotor disturbance, dysarthria, limb ataxia, horizontal and vertical supranuclear gaze palsy, urge incontinence, divergent strabismus, mild axonal sensory, neuropathy	Cerebellar > cortical atrophy, ear of the lynx sign	32/F	Bosnian	Estrada‐Cuzcano et al. (2017)
c.2629G>A	p.Gly877Arg	H	Spastic gait, hyperreflexia, falls, bilateral adductor response of knee jerk, pyramidal hypertonia, questionable bradykinesia, dysdiadochokinesia, balance difficulties, ocular disturbances, slurred speech, mental retardation, Babinski signs, brisk reflexes, slightly increased axial and appendicular tone, mild parkinsonism	Generalized atrophy	31/M	Italy	Erro et al. (2019)
c.2473_2474insAAdelC	p.(Leu825Asnfs*32)	H	Spastic paraplegia, lower extremity spasticity, lower extremity hyperreflexia, Babinski signs, spinocerebellar ataxia, dysarthria, falls, swallowing difficulty, cognitive decline, urinary complications, sensory abnormalities, mild upper extremity hyperreflexia, slow and ataxic saccades, dysdiadochokinesia, bucco‐lingual dyskinesias, bradykinesia, action and Parkinson tremor, dysmetria, behavior problems	Diffuse cerebellar atrophy	31/F	Inuit Canadian	Present Study
c.2126G>C	p.(Arg709Thr)	H	Spastic paraplegia, lower extremity hyperreflexia, lower extremity spasticity, Babinski sign, upper extremity hyperreflexia, dysarthria, fine motor impairment, learning difficulty, mild intellectual disability, cognitive decline, atrophy, pes cavus, mild vibratory loss, seizures, mild nystagmus, saccadic pursuit, slow and ataxic saccades, fatigable right beating nystagmus, ataxic gait, delusions, hallucinations, bradykinesia, dysdiadochokinesia	Diffuse cerebral and cerebellar atrophy and hypoplasia of the corpus callosum.	25/M	Armenia‐Lebanon	Present Study
c.2158G>T	p.(Gly720Trp)	H	Spastic paraplegia, lower extremity spasticity, lower extremity weakness, hearing difficulty, learning difficulty, falls, Babinski signs, ataxia, ankle clonus, dysarthria, dysphagia, saccadic pursuit, psychotic episodes, paranoid delirium, no parkinsonism	Cortical and cerebellar atrophy with signs of leukoencephalopathy in semioval centers, especially on the right side	29/M	French‐Canadian	Present Study

Abbreviations: AA, amino acid; C, compound heterozygous; F, female; H, homozygous; M, male; MRI, magnetic resonance imaging.

#### 3.2.1 Patient A

**Figure 2:**
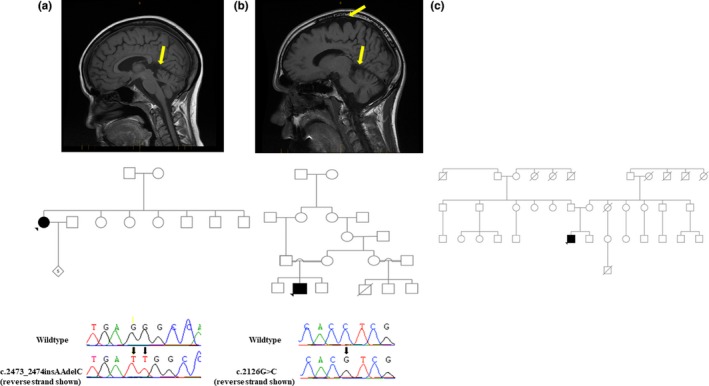
MRI images, pedigrees, and Sanger sequencing chromatograms. (a) Patient A’s MRI showed diffuse cerebellar atrophy (arrow). (b) Patient B’s MRI showed moderate diffuse cerebral and cerebellar atrophy (arrows). (c) MRI and DNA for sanger sequencing were not available for patient C

The patient, a 44‐year‐old woman of Inuit‐Canadian origin, was initially evaluated at age 31 due to gait dysfunction. She was found to have bilateral lower extremity spasticity, weakness, hyperreflexia, nonsustained bilateral ankle clonus and speech difficulties. On evaluation at age 40, she was laughing excessively and seemingly had an inappropriate affect. Minor Parkinsonian tremor and action tremor were noted. At age 43, the patient was agitated and verbally and physically aggressive. Fine movements were decreased, and spinocerebellar ataxia and prominent spastic paraplegia were present. Some of her parkinsonian symptoms may be attributed to her treatment with haloperidol. Brain and spine MRI demonstrated diffuse cerebellar atrophy and normal spine (Figure 2a). WES revealed a p.(Leu825Asnfs*32) mutation which results in a truncated peptide of 857 a.a and deletion of six C‐terminally located transmembrane alpha‐helixes. The mutation has not been reported in gnomAD and ExAC, but was previously reported (also as homozygous) in a patient with KRS from a Greenlandic Inuit family (Eiberg et al., 2012). As our patient is of Inuit‐Canadian family, this might suggest that this is an old, founder Inuit mutation.

#### 3.2.2 Patient B

During childhood, this patient from an Armenian‐Lebanese consanguineous family had impairment of fine motor movements, and experienced learning difficulties and delayed mental development noticeable at the age of 6 years. On evaluation at age 18, the patient presented with increased muscle tone especially in the lower extremities and gait was spastic. A Levodopa trial did not result in any improvement. At age 24, the patient had a seizure for the first time. MRI demonstrated moderate diffuse cerebral and cerebellar atrophy (Figure 2b). EEG demonstrated mild, slow biposterior dysfunction but without epileptiform patterns. On evaluation at the age of 31 years, the patient started to develop ideas of reference and delusions. WES was performed and identified a novel homozygous missense *ATP13A2* mutation, p.(Arg709Thr), in exon 19 within the hydrolase domain. The mutation is predicted to be deleterious by CADD (25), Polyphen‐2 (0.99), MutationTaster (1), and was located in a highly conserved amino acid with GERP++ score of 5.

#### 3.2.3 Patient C

At age 6, after normal development, this male patient of French‐Canadian origin was reported to have learning difficulties that became more pronounced through high school. At age 12, the patient started abusing alcohol and drugs, and throughout his teenage years he had two psychotic episodes and paranoid delusions. On evaluation at age 32, the patient had presented with spasticity and ataxia, spastic and mildly magnetic gait with frequent falls. Brain MRI done at the age of 29 showed cortical and cerebellar atrophy (images are not available). Metabolic workup, EEG, EMG, nerve conduction studies, and an abdominal ultrasound were normal. Clinical WES identified a homozygous *ATP13A2* missense mutation in exon 20, p.(Gly720Trp), predicted to be deleterious by SIFT (0), Polyphen‐2 (1), and CADD (31).

## 4 DISCUSSION

We describe three unrelated patients with predominant spastic paraplegia features, harboring homozygous *ATP13A2* mutations that are either novel or were not previously reported in HSP. *ATP13A2*‐HSP is rare, responsible for 0.4% of all HSP patients and 0.7% of all HSP families in CanHSP. Interestingly, all three patients suffered from psychiatric symptoms, which were previously reported in only one SPG78 patient (Estrada‐Cuzcano et al., 2017). One of the patients has developed seizures, which have not been previously reported in SPG78. Mild extrapyramidal symptoms/signs were present in patient A and bradykinesia in patient B. MRI in all three patients demonstrated cerebellar and/or cerebral atrophy, consistent with previous reports on *ATP13A2‐*HSP (Table 1).

Interestingly, the p.(Leu825Asnfs*32) mutation in patient A resulted in HSP‐predominant phenotype, while in previous patients reported with the same mutation it was Parkinsonism‐dominant phenotype (Eiberg et al., 2012). This may suggest that the clinical presentation may be affected by other genetic and/or environmental factors. We also identified a novel missense *ATP13A2* variant, p.(Arg709Thr) in a highly conserved amino acid located within the hydrolase domain which is critical for the catalytic activity of ATP13A2. Furthermore, this variant is affecting the last nucleotide of the exon, which may also affect splicing and possibly result in nonsense mediated decay. This possibility needs to be studied preferably in neuronal models with the variant. The mutation in patient C, p.(Gly720Trp) changes Glycine to Tryptophan at codon 720, which could unfold the N‐domain of ATP13A2 (Figure 1e). Our pathway enrichment analysis may suggest that copper ion binding is involved in the pathogenesis of specific forms of HSP, and further studies are required to examine this possibility.

This study has several limitations. Since DNA was not available for segregation analysis, and since our genetic data include only in silico prediction tools and structural models, we could not prove with full confidence that the detected variants in *ATP13A2* are disease causing. However, one of the mutations was previously described in a patient, and it is unlikely that by chance alone two extremely rare biallelic variants in a gene that is already known as disease causing will be found in two HSP patients. Therefore, it is probable that these variants are disease causing. An additional limitation is the lack of available DNA for patient C, therefore the variant reported by the clinical lab could not be independently confirmed.

Our study expands the genetic and phenotypic spectrum of *ATP13A2*‐related HSP. The different phenotypes observed in carriers of *ATP13A2* mutations imply that genetic and nongenetic modifiers exist. Our findings may also suggest that in HSP patients with psychiatric symptoms, mutations in *ATP13A2* should be suspected, especially if mild parkinsonian symptoms are also present.

## CONFLICT OF INTERESTS

The authors declare no conflicts of interest.

## AUTHORS’ CONTRIBUTIONS

MAE, GAR, and ZG‐O conceived the study design, MAE, EL, DS, ND, and JFT performed data analysis MAE and ZG‐O wrote the paper. All authors have read, edited, and approved the final version of the manuscript.

## Data Availability

All data reported here are available upon request.
